# Long-Term Outcome of Patients with Low-Risk Differentiated Thyroid Cancer Treated with Total Thyroidectomy Alone

**DOI:** 10.3390/curroncol31090409

**Published:** 2024-09-16

**Authors:** Antonio Matrone, Alessio Faranda, Liborio Torregrossa, Carla Gambale, Elisa Minaldi, Alessandro Prete, Luigi De Napoli, Leonardo Rossi, Laura Agate, Virginia Cappagli, Luciana Puleo, Eleonora Molinaro, Gabriele Materazzi, Rossella Elisei

**Affiliations:** 1 Unit of Endocrinology, Department of Clinical and Experimental Medicine, Pisa University Hospital, 56124 Pisa, Italy; a.faranda@studenti.unipi.it (A.F.); carla.gambale@phd.unipi.it (C.G.); elisa.minaldi@phd.unipi.it (E.M.); alessandro.prete@phd.unipi.it (A.P.); l.agate@ao-pisa.toscana.it (L.A.); virginia.cappagli@med.unipi.it (V.C.); luciana.puleo@med.unipi.it (L.P.); e.molinaro@ao-pisa.toscana.it (E.M.); rossella.elisei@unipi.it (R.E.); 2 Pathology Unit 3, Department of Surgical, Medical, Molecular Pathology and Critical Area, Pisa University Hospital, 56124 Pisa, Italy; liborio.torregrossa@unipi.it; 3 Unit of Endocrine Surgery, Department of Surgical, Medical, Molecular Pathology and Critical Area, Pisa University Hospital, 56124 Pisa, Italy; l.denapoli@ao-pisa.toscana.it (L.D.N.); leonardo.rossi@phd.unipi.it (L.R.); gabriele.materazzi@unipi.it (G.M.)

**Keywords:** low-risk thyroid cancer, postoperative management, remnant ablation, radioiodine treatment, biochemical events, papillary thyroid cancer, active surveillance, thyroglobulin, thyroglobulin antibodies

## Abstract

Background: Differentiated thyroid carcinoma (DTC), mainly papillary (PTC), at low risk of recurrence is currently managed with active surveillance strategies or less aggressive surgeries. However, total thyroidectomy with ^131^I treatment is still performed both if these tumors are diagnosed before or occasionally after surgery. This real-life study aimed to evaluate the rate of biochemical, structural, and functional events in a large series of consecutive DTCs at low risk of recurrence treated by total thyroidectomy, but not with ^131^I, in a medium–long-term follow-up. Patients and Methods: We evaluated clinical–pathologic data of 383 consecutive patients (2006–2012) with unifocal DTC [T1a/b(s)] at low risk of recurrence, treated with total thyroidectomy but without lymph node dissection and ^131^I treatment after surgery. We evaluated if structural, biochemical, and functional events were detected during the follow-up. Results: Females accounted for 75.7% of our study group, and the median age was 50 years. The median tumor dimension was 0.4 cm (range 0.1–1.2). Most of the patients had a unifocal T1a tumor (98.9%), and 73.6% had a classic variant of PTC. We divided the patients according to the absence (group A—n = 276) or presence (group B—n = 107) of interfering TgAb at first control after surgery. After a median follow-up of 10 years, no structural events were detected. Sixteen out of three hundred and eighty-three (4.2%) patients developed biochemical events: 12/276 (4.3%) in group A and 4/107 (3.7%) in group B. The median time elapsed from surgery to detecting a biochemical event was 14.5 and 77.5 months in groups A and B, respectively. No patients performed additional treatments and were followed up with an active surveillance strategy. Conclusions: This study confirmed that patients with DTC at low risk of recurrence showed an excellent outcome in a medium long-term follow-up since no structural events were diagnosed. Significant variations in Tg/TgAb were detected in a few cases, all managed with an active surveillance strategy without the need for other treatments. Therefore, a relaxed follow-up with neck ultrasound and Tg/TgAb measurement is enough to early identify those very unusual cases of recurrence.

## 1. Introduction

Thyroid cancer is the most common malignant neoplasm of the endocrine system. In the last 20–25 years, its incidence has significantly increased [1,2], mainly due to the detection, often occasionally, of small thyroid papillary carcinomas (PTCs) [3]. Despite this increased incidence, mortality has remained stable over time [4].

Total thyroidectomy, followed by radioiodine treatment (^131^I), was historically the initial treatment of choice for all stages of differentiated thyroid carcinoma (DTC). However, in recent years, the increasing knowledge about the clinical, histologic, and molecular features of DTC, either before or after surgery, has allowed for clinicians to better select patients who could benefit from a less aggressive initial treatment. Particularly, in PTC ≤ 1 cm a de-escalation therapeutic approach was applied over time, passing by total thyroidectomy alone without ^131^I [5] to lobectomy (with or without isthmectomy) [6,7] up to active surveillance programs [8,9].

Regarding the postoperative treatment with ^131^I, two large non-inferiority clinical trials published in 2012 (HiLo [10] and ESTIMABL1 [11]) showed that in patients with low-risk DTC, the use of low activities of ^131^I (1.1 GBq) showed similar results at 6–9 months when compared with high activities (3.7 GBq). Moreover, these results were confirmed in the two follow-up studies derived from the two clinical trials mentioned above, with a longer median follow-up of 6.5 years [12] and 5.4 years, respectively [13].

Recently, the ESTIMABL2 trial [14] showed non-inferiority, in a 3-year follow-up of the strategy of “not performing ^131^I” compared to using low activity of ^131^I (1.1 GBq), in patients with low-risk DTC. The study has been well accepted but criticized for the relatively short follow-up [15].

In our institution, from 2006, according to the European consensus for the management of patients with differentiated thyroid carcinoma of the follicular epithelium [5], patients with single DTC ≤ 1 cm did not perform the ^131^I postoperative remnant ablation anymore.

This study aimed to evaluate the rates of biochemical, structural, and functional events in a large group of DTC patients at low risk of recurrence, treated by surgery but not with ^131^I, during a medium–long-term follow-up.

## 2. Patients and Methods

From a prospectively maintained database, we retrospectively evaluated epidemiological, clinical, and pathological data of a consecutive series of patients with DTC at low risk of recurrence as defined by the 2009 ATA guidelines [16]. All patients were treated, according to the indications of that time, with total thyroidectomy between 2006 and 2012, without prophylactic or therapeutic lymph node dissection, and followed at the Unit of Endocrinology of the University Hospital of Pisa.

The group consisted of 383 consecutive patients with unifocal DTC [T1a/b(s)] who, according to the 2006 European Consensus for the management of patients with DTC [5], were not submitted to postoperative treatment with ^131^I. All patients had at least 3 clinical, biochemical, and ultrasonographic evaluations, performed in our department.

### 2.1. Thyroglobulin (Tg) and Thyroglobulin Antibodies (TgAb) Measurement

A highly ultrasensitive chemiluminescent assay (Beckman Coulter, Fullerton, CA, USA, with a functional sensitivity of 0.1 ng/mL) was used for serum Tg measurement. Serum TgAb was analyzed by a Fluorescence Enzyme Immuno Assay (AIA-Pack 2000; Tosoh Corporation, Tokyo, Japan). For TgAb, the recommended manufacturer value able to distinguish the thyroid autoimmune disease in the general population with a thyroid gland in situ was 30 IU/mL. The analytical sensitivity, which refers to the precision of the zero matrix, was 6 IU/mL according to the manufacturer and 1 IU/mL according to our laboratory. However, as established from the 20% between-run CV [17,18], the lower limit of TgAb titer not interfering with the Tg assay was 8 IU/mL.

### 2.2. Neck Ultrasound (Neck US)

Neck US was performed using a 7.5–12 Mhz multifrequency linear probe by a physician with at least 5 years of experience in this imaging method. During the study period, different color Doppler types of equipment were used: from 2006 to 2012 AU 590 Asynchronous (EsaoteBiomedica, Firenze, Italy), from 2013 to 2018 MyLab 50 (EsaoteBiomedica, Florence, Italy), and since 2019, MyLab Twice (EsaoteBiomedica, Florence, Italy). Ultrasonographic suspicious lymph nodes were evaluated by FNAC and Tg assay on washing fluid.

### 2.3. Follow-Up Strategy

After surgery, all patients performed a regular follow-up over time with the first examination 4–6 months after surgery and every 12–24 months thereafter. Blood evaluation for TSH, Tg and TgAb, and neck US were performed at each clinical evaluation. Further imaging procedures (CT scan, MRI, 18FDG-PET scan, etc.) were performed, if needed, following the suggestions of the guidelines in force at the time of the follow-up [5,6,16].

For the aim of this study, at each clinical evaluation, we defined the following:

(1) Structural events: if abnormal lesions were detected at the neck US and were cytologically confirmed with fine needle aspiration and/or the presence of distant metastatic disease detected by other imaging methods.
(2) Biochemical events: we distinguished two possible scenarios according to the presence or absence of interfering serum TgAb: (a) in TgAb-negative patients (TgAb ≤ 8 IU/mL), when LT4-Tg values were >5 ng/mL once during follow-up or >2 ng/mL in two consecutive evaluations or when a de novo appearance of TgAb > 8 IU/mL was observed; (b) in TgAb-positive patients (TgAb > 8 IU/mL), when there was an increase in TgAb values > 50% of the previous value in at least two consecutive evaluations.

### 2.4. Statistical Analysis

Categorical variables are expressed as counts and frequency. The Kolmogorov–Smirnov test was used to assess the normality of data. Continuous variables are expressed as mean and standard deviation or median and interquartile range, according to the distribution of the data. Statistical analysis was performed by SPSS (version 20.0, Armonk, NY, USA: IBM Corp).

## 3. Results

In our study group, 75.7% of patients were females and the median age was 50 years (IQR 40.75–59, range 12–75). The median tumor dimension was 0.4 cm (IQR 0.2–0.6, range 0.1–1.2). Most patients had a unifocal T1a tumor (98.9%). Regarding histologic variant, 73.6% of patients had classic variant PTC (CV-PTC), 23.7% follicular variant PTC (FV-PTC), 2.4% aggressive variants of PTC (AV-PTC—six tall cell variants and three solid variants), and one case was diagnosed as follicular thyroid carcinoma (FTC).

The epidemiological and clinical–pathological data of the whole study group are shown in Table 1.

According to the values of TgAb at the first postoperative evaluation, we divided patients into two groups as follows: group A (TgAb-negative [<8 UI/mL]: 276/383 patients—72%) and group B (TgAb-positive [>8 UI/mL]: 107/383 patients—28%).

Patients were followed up for a median time of 10 years (IQR 6.1–13.5, range 2–17), performing a median of 6 (IQR 4–7, range 3–15) clinical, biochemical, and neck US evaluations for each patient over time. During the follow-up, no structural or functional events were recorded. Overall, 16/383 (4.2%) patients showed the onset of a biochemical event. The median time elapsed from surgery to the detection of the biochemical event was 17 months (IQR 8.5–62.5, range 3–127).

### 3.1. Biochemical Events in Group A (TgAb-Negative) and Group B (TgAb-Positive)

When classifying these events according to group A or B, 12/276 (4.3%) were in group A and mainly due to an increase in Tg values (n = 11), while only one event was due to the de novo appearance of TgAb in a patient with slightly detectable Tg values. When we evaluated these patients in detail, we noticed that in two cases (patients #13 and #15), the increase in Tg values was linked to the simultaneous presence of higher TSH values, concerning the previous ones, due to low compliance of the patients to the treatment. Indeed, when the L-T4 dosage was adjusted and the TSH returned to the normal range, Tg values significantly decreased (Table 2) coming back to the previous values. Conversely, 4/107 (3.7%) biochemical events were detected in group B due to a continuously increasing trend over time of TgAb values (Table 3).

The median time elapsed from surgery to detecting a biochemical event was 14.5 months (IQR 5.5–47.75, range 3–105) and 77.5 months (IQR 52.75–119.5, range 15–127) in groups A and B, respectively.

We also looked for epidemiologic (age and sex), clinical (modality of diagnosis, preoperative or at histology), or pathologic features (tumor dimension, histologic variant) potentially correlated with biochemical events, but no significant correlation was found.

### 3.2. Outcome

No patients, including those with the appearance of biochemical events, showed positive neck US and/or further imaging when performed. For this reason, all patients were maintained on active surveillance without performing any additional treatment (i.e., surgeries or treatment with ^131^I). At the time of the data lock of this study, after a median follow-up time of 10 years, all patients who experienced biochemical events had not develop any structural disease yet.

## 4. Discussion

To date, the management of patients with low-risk DTC is moving towards a more conservative approach, from less invasive initial treatments to active surveillance [19,20]. In particular, many authors agree about the lack of evidence that a postoperative remnant ablation with ^131^I in low-risk DTC could produce any benefit in terms of survival and recurrence [5,6,15]. However, several cases are still treated with total thyroidectomy [21] and, in some of them, ^131^I treatment is also inappropriately performed [22]. Moreover, it is conceivable that some of these tumors can derive from incidental findings in histology due to surgical treatment of total thyroidectomy for other reasons (i.e., Graves’ disease, multinodular goiter) [23]. In this regard, the postoperative management of these tumors, particularly in cases with detectable Tg or TgAb, is still disputable and the use of ^131^I treatment to ablate the postoperative remnant and facilitate the follow-up, also in low-risk DTC, is still sustained by some authors [24]. Despite some criticism related to the inclusion criteria (i.e., patients with undetectable/not measured Tg before randomization, relatively low ^131^I activity) but mainly to the relatively short follow-up (i.e., 3 years) [15,25,26], the prospective and randomized ESTIMABL 2 study clearly demonstrated that in low-risk patients this procedure is not more useful [14]. Although our study is not prospective nor randomized, patients were followed up for a longer time (i.e., the median time of 10 years with a range of up to 17 years) during which the clinical and biochemical data were prospectively collected.

In our study, none of the 383 consecutive patients with low-risk, mainly unifocal DTC, without lymph node metastases who were treated with total thyroidectomy but without ^131^I, showed a structural recurrence during this relatively long-term follow-up. Similar data were shown in previous studies in which the recurrence rate of low-risk patients, treated by total thyroidectomy but without ^131^I, was very low ranging from 0 to 1.6% [27,28,29,30,31,32]. It is worth noting that in the ESTIMABL2 study, few structural events were recorded, all characterized by metastatic lymph nodes. However, no differences in the lymph node recurrences between the group of patients treated with (n = 2) or without (n = 2) postoperative ^131^I were highlighted [14]. The absence of structural events in our series could be explained by the different histologic features of our study group, which included patients with mostly unifocal tumors and smaller in size, compared with the ESTIMABL2 study. Indeed, this finding is not surprising since nowadays, according to Japanese studies [8,33], small papillary thyroid cancer (i.e., microPTC) grows very rarely and only very few cases develop lymph nodes but not distant metastases. According to these findings, not only does postoperative remnant ^131^I ablation have no benefits in terms of recurrence and survival, but even immediate surgery should be avoided in most of these cases.

Despite no structural events being observed, we experienced 16/383 (4.2%) biochemical events whose rate was similar in the two groups A and B. Our data are in line with those shown in the ESTIMABL2 study [14] in which the rate of biochemical events in DTC not treated with ^131^I was 3.8%. Regarding the biochemical events and in particular Tg increase, we should be aware that Tg values, even at this low level, are highly dependent on TSH [34], and before making therapeutic decisions, Tg values should be rechecked over time in the presence of comparable TSH values, possibly in the normal ranges. Only in cases of persistent increasing trend of Tg values, with constants and comparable TSH values, should further diagnostic or therapeutic strategies be considered [35]. Moreover, since the postsurgical remnant has not been ablated, an increase in serum Tg could be due to the presence of normal thyroid remnant tissue and active surveillance should be the preferred management [20,34,36,37].

Biochemical events due to the increase in TgAb were detected in our series at the same rate as those due to Tg increase (4/107—3.7%). Since a transient increase in TgAb could be due to an activation of the immune system due to several events such as inflammations or infections [38], in these cases, the trend of TgAb titer over time, rather than the single increased value, should also be monitored. Moreover, both in cases treated [39] or not with ^131^I [18,40], the rate of decrease in TgAb over time is influenced by several factors, and not all patients exhibited the same rate of decline.

In a single patient (0.4%) we observed a de novo appearance of TgAb during the follow-up, with a peak of 38 IU/mL 2 years after the surgery. However, a spontaneous decrease of TgAb over time was observed up to becoming undetectable (<1 IU/mL) 7 years after surgery. In this period, the patient was followed up, no structural disease was observed, and no active therapies were performed.

Regarding the biochemical events, no predictive epidemiologic, clinical, and/or pathological features were found. This result is of clinical interest mainly considering that, although small, a percentage of cases in our series (2.8%) had an aggressive histologic variant that is still considered a feature upgrading from low to intermediate risk of recurrence [6]. This evidence strongly suggests that small DTCs, with no clinical and ultrasonographic evidence of lymph node metastases, belong to the low-risk category independently from any other feature. Moreover, in our institution, prophylactic central compartment neck dissection is not routinely performed and none of the patients included in the study group underwent this procedure. The absence of structural events in a medium–long term follow-up indirectly demonstrated that in low-risk DTC, without preoperative evidence of lymph node metastases at the neck US, not having performed prophylactic central compartment lymph node dissection did not impact the clinical outcome of these patients.

Despite the limitation due to the retrospective nature of this study, although the data were prospectively collected, the main strengths are represented by the long-term follow-up and the uniform mode of management of the patients at the same institution, with the same clinical, biochemical, and imaging method for all the duration of the follow-up.

## 5. Conclusions

In conclusion, the results of this study showed an excellent outcome for patients with low-risk DTC treated with total thyroidectomy and not ^131^I in a relatively long-term follow-up. No structural recurrence was observed, and in the few cases of biochemical recurrence, a “wait and see” strategy allowed for avoiding overtreatment and to verify that none of the patients developed structural disease. Therefore, it may be suggested by the findings of the present study that low-risk DTC, whenever treated with total thyroidectomy, does not receive any benefit from further treatment with ^131^I and can be considered cured by surgery alone. In the same context, they may not need a too close follow-up but rather clinical evaluations with neck ultrasound and Tg and TgAb measurements every 12–18 months to identify those very unusual cases of recurrence.

## Figures and Tables

**Table 1:** Epidemiologic and pathologic data of the study group (n = 383).

Gender	M	93 (24.3%)
	F	290 (75.7%)
Age	Median (IQR) [range]	50 years (40.75–59) [12–75]
Histology	CV-PTC	282 (73.6%)
	FV-PTC	91 (23.7%)
	AV-PTC	9 (2.4%)
	FTC	1 (0.3%)
Tumor size	Median (IQR) [range]	0.4 cm (0.2–0.6) (0.1–1.2)

Abbreviations: CV-PTC: classic variant papillary thyroid carcinoma; FV-PTC: follicular variant papillary thyroid carcinoma; AV-PTC: aggressive variant papillary thyroid carcinoma; FTC: follicular thyroid carcinoma.

**Table 2:** Details about patients with biochemical events in group A (TgAb-negative).

ID	Age at Surgery (yrs)	Gender	Histology	Tumor Size (cm)	Type of Event	Time Elapsed from Diagnosis to Event (months)	Tg Event (ng/mL)	TSH Event (mUI/L)	Last Tg (ng/mL)	Last TSH (mUI/L)	Tg Trend from Event to Last Evaluation	Follow-Up Time (months)
5	47	M	AV-PTC *	0.2	Tg > 5 ng/mL	17	5	3.96	0.57	0.144	Decrease	92
6	60	M	FV-PTC	0.7	Tg > 5 ng/mL	5	8.4	4.73	1.46	0.069	Decrease	50
7	65	F	FV-PTC	0.6	Tg > 5 ng/mL	3	7.1	1.74	2.47	0.308	Decrease	19
11	56	M	CV-PTC	0.6	Tg > 5 ng/mL	7	5.02	0.133	5.19	0.217	Stable	126
13	27	F	CV-PTC	0.6	Tg > 5 ng/mL	5	6.7	59.6	0.26	0.626	Decrease	37
9	59	F	FV-PTC	0.2	2 × Tg > 2 ng/mL	64	3.3	3.1	3.21	2.97	Stable	88
10	42	M	CV-PTC	0.1	2 × Tg > 2 ng/mL	58	4.4	0.232	3.01	0.005	Decrease	100
12	48	M	CV-PTC	0.4	2 × Tg > 2 ng/mL	105	3	0.417	4.22	0.257	Increase	137
14	31	F	CV-PTC	0.7	2 × Tg > 2 ng/mL	17	2.7	0.591	1.15	2.68	Decrease	64
15	71	F	CV-PTC	0.2	2 × Tg > 2 ng/mL	14	3.03	8.68	0.46	2.23	Decrease	141
16	49	F	CV-PTC	0.3	2 × Tg > 2 ng/mL	13	3.78	0.81	2.84	0.473	Decrease	139

Abbreviations: CV-PTC: classic variant papillary thyroid carcinoma; FV-PTC: follicular variant papillary thyroid carcinoma; AV-PTC: aggressive variant papillary thyroid carcinoma; FTC: follicular thyroid carcinoma. * tall cell variant.

**Table 3:** Details about patients with biochemical events in group B (TgAb-positive).

ID	Age at Surgery (yrs)	Gender	Histology	Tumor Size (cm)	Type of Event	Time Elapsed from Diagnosis to Event (months)	AbTg Event (UI/mL)	Tg Event (ng/mL)	TSH Event (mUI/L)	Last AbTg (UI/mL)	Last Tg (ng/mL)	Last TSH (mUI/L)	AbTg Trend from Event to Last Evaluation	Follow-Up Time (months)
2	43	F	CV-PTC	0.7	AbTg > 50%	97	26	0.1	2.21	26	0.1	2.21	Stable	97
3	56	F	CV-PTC	0.3	AbTg > 50%	58	17	0.31	0.28	58	0.27	0.282	Increase	127
4	54	F	CV-PTC	0.2	AbTg > 50%	127	14	0.01	0.05	14	0.01	0.05	Stable	127
1	75	F	CV-PTC	0.1	AbTg > 50%	51	9,2	0.12	1.20	13	0.01	0.55	Stable	64
8	61	F	FV-PTC	0.4	AbTg appearance	15	38	0.01	0.06	0,1	0.01	0.07	Decrease	180

Abbreviations: CV-PTC: classic variant papillary thyroid carcinoma; FV-PTC: follicular variant papillary thyroid carcinoma; AV-PTC: aggressive variant papillary thyroid carcinoma; FTC: follicular thyroid carcinoma.

## Data Availability

The data presented in this study are available on request from the corresponding author due to ethical reasons.
